# Supplementary material for: High-sensitivity cardiac troponin I and risk of dementia: the 25-year longitudinal Whitehall II study
Source: Eur Heart J. 2025 Nov 6;47(3):359–69. doi: 10.1093/eurheartj/ehaf834 (PMC12807566; doi:10.1093/eurheartj/ehaf834)
Supplement: ehaf834_Supplementary_Data [file ehaf834_supplementary_data.docx]

**Supplementary Materials**

**High-sensitivity cardiac troponin I and risk of dementia: 25-year longitudinal study in the Whitehall II cohort**

Yuntao Chen^ab^, Martin Shipley^a^, Atul Anand^c^, Dorien M Kimenai^c^, Klaus P Ebmeier^d^, Severine Sabia^ee^, Archana Singh-Manoux^e^, John Deanfield^f^, Mika Kivimaki^a^, Gill Livingston^a^, Nicholas L Mills^cg^, Eric J Brunner^b^

a Division of Psychiatry, University College London, London, UK

b Department of Epidemiology and Public Health, University College London, London, UK

c British Heart Foundation Centre for Cardiovascular Science, University of Edinburgh, Edinburgh, UK

d Department of Psychiatry and Wellcome Centre for integrative Neuroimaging, University of Oxford, Oxford, UK

e Inserm U1153, Epidemiology of Ageing and Neurodegenerative diseases, Universite de Paris, Paris, France

f Institute of Cardiovascular Science, University College London, London, UK

g Usher Institute, University of Edinburgh, Edinburgh, UK

Correspondence to:

Yuntao Chen

Division of Psychiatry, University College London, London, UK

Maple House, 149 Tottenham Court Road, London, W1T TNF

Email address: [yuntao.chen@ucl.ac.uk](mailto:yuntao.chen@ucl.ac.uk)

Equations for random slope linear mixed models:

${Cognitive}_{ij}=\beta_{0}+\beta_{1}{age}_{ij}+\beta_{2}{age}_{ij}^{2}+\beta_{3}{Troponin}_{i}+\beta_{4}Z_{i}+\beta_{5}{{Troponin}_{i}age}_{ij}+\beta_{6}{Troponin}_{i}{age}_{ij}^{2}+\beta_{7}{Z_{i}age}_{ij}+\beta_{8}Z_{i}{age}_{ij}^{2}+\mu_{0i}+{\mu_{1i}age}_{ij}{+\mu}_{2i}{age}_{ij}^{2}+\varepsilon_{ij}$,

where ${Cognitive}_{ij}$ is the cognition score of the $i^{th}$ participant at the $j^{th}$ occasion; ${age}_{ij}$ is the age (centred at 65 years) of the $i^{th}$ participant at the $j^{th}$ occasion; ${Troponin}_{i}$ is the log2 transformed cardiac troponin level for the $i^{th}$ participant at baseline; $Z_{i}$ is the vector of time independent covariates (sex, education, occupational position, birth cohort) for the $i^{th}$ participant at baseline; $\mu_{0i}$ is the random intercept; $\mu_{1i}$ is the random slope for linear age effect; $\mu_{2i}$ is the random slope for the quadratic age effect; $\varepsilon_{ij}$ is the residual,

with $\left[ \begin{matrix} \mu_{0i} \\ \mu_{1i} \\ \mu_{2i} \end{matrix} \right]\sim N(\left[ \begin{matrix} 0 \\ 0 \\ 0 \end{matrix} \right],\boldsymbol{D})$, and $\varepsilon_{ij}\sim N(0,\sigma^{2})$,

where $\boldsymbol{D}$ is an unstructured $3\times3$ variance-covariance matrix allowing correlation among intercept, slope, and quadratic terms. The random effects and error term are both assumed normally distributed, and the error term is assumed independent of the random effects.

We checked the normality assumption for the linear mixed model using the Q-Q plot. The points mostly followed the straight diagonal line in the middle portion, suggesting that the residuals were approximately normally distributed.


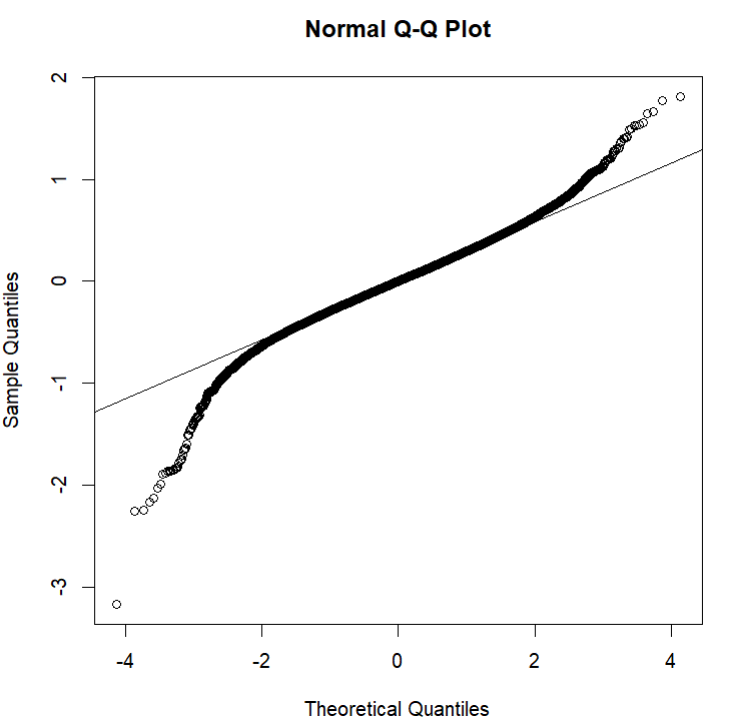


We checked the homoscedasticity assumption using the residual plot below where we plotted the residuals across fitted values from the mixed model. The residuals generally scattered around the 0 line, suggesting homoscedasticity.


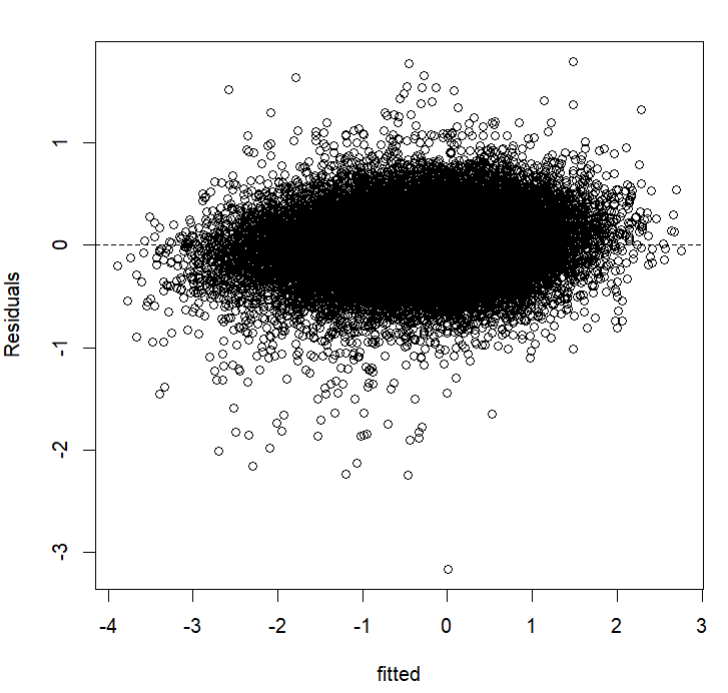


Table S1 Association between high-sensitivity cardiac troponin I at baseline (1997-99) and incident dementia during 25 years of follow-up in a subsample with data on APOE genotype (N=4562)

| **Cardiac troponin I, ng/L** | **No of cases/total No** | **HR (95% CI)** | **P value** |
| --- | --- | --- | --- |
| <2.5 | 109/1614 | 1.00 (reference) |  |
| 2.5-3.4 | 98/996 | 1.27 (0.96,1.68) | 0.095 |
| 3.5-5.2 | 110/982 | 1.33 (1.00,1.76) | 0.054 |
| >5.2 | 115/970 | 1.40 (1.05,1.86) | 0.022 |

The model was adjusted for age, sex, ethnicity, education, occupational position, smoking status, alcohol consumption, physical activity, BMI, systolic blood pressure, diastolic blood pressure, glucose, total cholesterol, triglycerides, high-density lipoprotein cholesterol, anti-hypertensive drugs, diabetes medication, lipid lowering drugs and APOE ε4 (0, 1 or 2) at baseline.

Table S2 Association between high-sensitivity cardiac troponin I at baseline (1997-99) and incident dementia during 25 years of follow-up in a subsample with data on eGFR at 2007-09 (N=4829)

| **Cardiac troponin I, ng/L** | **No of cases/total No** | **HR (95% CI)** | **P value** |
| --- | --- | --- | --- |
| <2.5 | 112/1722 | 1.00 (reference) |  |
| 2.5-3.4 | 91/1051 | 1.11 (0.83,1.48) | 0.483 |
| 3.5-5.2 | 109/1049 | 1.22 (0.92,1.61) | 0.170 |
| >5.2 | 130/1008 | 1.48 (1.12,1.94) | 0.005 |

The model was adjusted for age, sex, ethnicity, education, occupational position, smoking status, alcohol consumption, physical activity, BMI, systolic blood pressure, diastolic blood pressure, glucose, total cholesterol, triglycerides, high-density lipoprotein cholesterol, anti-hypertensive drugs, diabetes medication, lipid lowering drugs, and eGFR at phase 9 (2007-09).

Table S3 Association of high-sensitivity cardiac troponin I with cognitive performance at age 60, 70, 80, 90 years.

| **Difference in cognitive function** | | | | |
| --- | --- | --- | --- | --- |
|  | **Age 60** | **Age 70** | **Age 80** | **Age 90** |
| **Cardiac troponin I, ng/L** | | | | |
| <2.5 | ref | ref | ref | ref |
| 2.5-3.4 | 0 (-0.05,0.05) | -0.03 (-0.08,0.02) | -0.03 (-0.11,0.05) | 0.01 (-0.14,0.17) |
| 3.5-5.2 | -0.02 (-0.08,0.03) | **-0.05 (-0.11,0)** | -0.06 (-0.14,0.01) | -0.05 (-0.21,0.11) |
| >5.2 | 0.01 (-0.04,0.06) | -0.02 (-0.08,0.03) | **-0.09 (-0.17,-0.01)** | **-0.18 (-0.34,-0.02)** |

The model term included sex, ethnicity, education level, occupational position, birth cohort, cardiac troponin group, age, age2, and interactions of sex, ethnicity, education level, occupational position, birth cohort, cardiac troponin group with age and age2, alcohol consumption, smoking status, physical activity, BMI, SBP, DBP, glucose, total cholesterol, triglycerides, high-density lipoprotein cholesterol, anti-hypertensive drugs, diabetes medication, and lipid lowering drugs.

Table S4 Difference in cardiac troponin I level (ng/L) between dementia cases and controls in the years preceding dementia diagnosis. Nested case-control design matching with age, sex and education level.

| **Year** | **Difference in cardiac troponin I level*** |
| --- | --- |
| -25 | 0.56 (0.20,0.92) |
| -20 | 0.45 (0.18,0.72) |
| -15 | 0.35 (0.10,0.61) |
| -10 | 0.28 (0.04,0.51) |
| -5 | 0.22 (-0.02,0.46) |
| 0 | 0.18 (-0.19,0.54) |

*Values were calculated as averaged predicted difference of cardiac troponin level (log2 transformed) between cases and controls, and its 95% confidence interval. The latent process mixed model included time, time2, index age (age at time 0), sex, education level, case indicator (coded as 1 for cases and 0 for controls), and interactions of the covariates (index age, sex, education level, and case indicator) with time and time2.

Table S5 Difference in cardiac troponin I level (ng/L) between dementia cases and controls in the years preceding dementia diagnosis. Nested case-control design matching with age, sex and education level and cardiovascular disease status.

| **Year** | **Difference in cardiac troponin I level*** |
| --- | --- |
| -25 | 0.50 (0.13,0.86) |
| -20 | 0.40 (0.14,0.67) |
| -15 | 0.31 (0.06,0.56) |
| -10 | 0.22 (-0.01,0.45) |
| -5 | 0.14 (-0.10,0.37) |
| 0 | 0.05 (-0.31,0.41) |

*Values were calculated as averaged predicted difference of cardiac troponin level (log2 transformed) between cases and controls, and its 95% confidence interval. The latent process mixed model included time, time2, index age (age at time 0), sex, education level, case indicator (coded as 1 for cases and 0 for controls), and interactions of the covariates (index age, sex, education level, and case indicator) with time and time2,

Table S6 Difference in cardiac troponin I level (ng/L) between dementia cases and controls in the years preceding dementia diagnosis. Nested case-control design matching with age, sex and education level.

| **Year** | **Difference in cardiac troponin I level*** |
| --- | --- |
| -25 | 0.43 (0.05,0.81) |
| -20 | 0.41 (0.14,0.69) |
| -15 | 0.38 (0.13,0.62) |
| -10 | 0.31 (0.09,0.54) |
| -5 | 0.23 (0,0.46) |
| 0 | 0.12 (-0.24,0.49) |

*Values were calculated as averaged predicted difference of cardiac troponin level (log2 transformed) between cases and controls, and its 95% confidence interval. The latent process mixed model included time, time2, index age (age at time 0), sex, education level, case indicator (coded as 1 for cases and 0 for controls), and interactions of the covariates (index age, sex, education level, and case indicator) with time and time2, time-dependent SBP, fast glucose, BMI, total cholesterol, triglycerides, and HDL at three waves (1997-99, 2007-09, 2012-13).

Table S7 Association between high-sensitivity cardiac troponin I at phase 5 (1997-99) and structural brain volume 15 years later: Whitehall II imaging substudy.

| **Cardiac troponin I, ng/L** | **White matter hyperintensities (total Fazekas score, range 0-6)** | | **White matter hyperintensities volume (BIANCA) (% total intracranial volume)** | | **DTI-derived global White matter fractional anisotropy** | | **DTI-derived global White matter mean diffusivity (*10^-4^), mm²/s** | |
| --- | --- | --- | --- | --- | --- | --- | --- | --- |
|  | **No** | **Ratio of the score (95%CI)** | **No** | **Difference (95%CI)** | **No** | **Difference (95%CI)** | **No** | **Difference (95%CI)** |
| <2.5 | 218 | 1 (ref) | 213 | 0 (ref) | 209 | 0 (ref) | 209 | 0 (ref) |
| 2.5-3.4 | 142 | 0.97 (0.85,1.11) | 135 | 0.007 (-0.038,0.052) | 133 | -0.003 (-0.006,0.001) | 133 | 0.027 (-0.026,0.079) |
| 3.5-5.2 | 135 | 0.95 (0.83,1.09) | 133 | 0.019 (-0.027,0.064) | 128 | -0.002 (-0.006,0.002) | 128 | 0.018 (-0.035,0.072) |
| >5.2 | 141 | 0.98 (0.86,1.13) | 134 | 0.005 (-0.041,0.051) | 130 | -0.003 (-0.007,0.001) | 130 | 0.042 (-0.012,0.096) |
| Every doubling in cardiac troponin I | 636 | 0.99 (0.94,1.03) | 615 | 0 (-0.02,0.01) | 600 | -0.001 (-0.002,0) | 600 | 0.013 (-0.004,0.030) |

For white matter hyperintensities (BIANCA), fractional anisotropy, and mean diffusivity, linear regression models were used adjusted for age, sex, ethnicity, education level, occupational position, smoking status, alcohol consumption, and magnetic resonance imaging scanner.

For white matter hyperintensities (Fazekas score), Poisson regressions were used adjusted for age, sex, ethnicity, education level, occupational position, smoking status, alcohol consumption, and magnetic resonance imaging scanner.


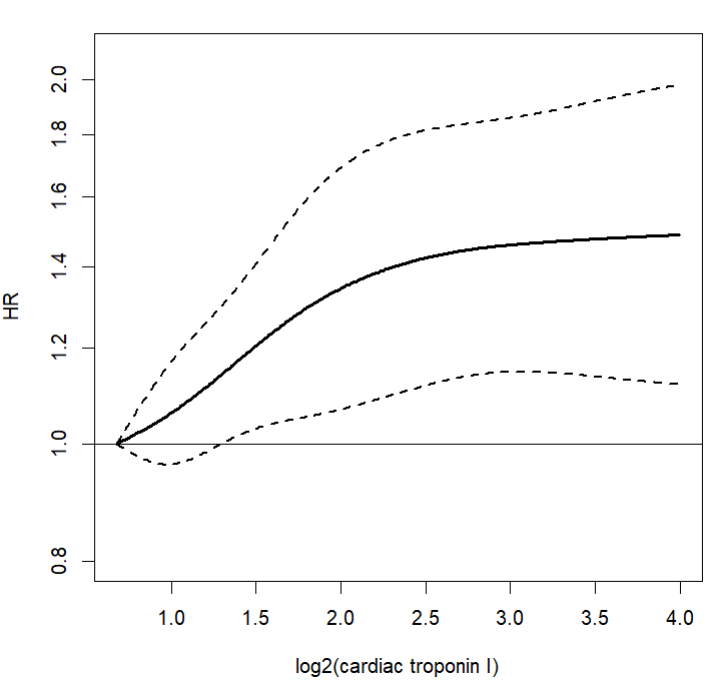


Figure S1 Association of high-sensitivity troponin I with incident dementia. Results from Cox model with spline function of high-sensitivity troponin I (knots at 10^th^, 50^th^, 90^th^ percentile in the population with high-sensitivity troponin I above detection limit) after adjusting for age (time scale), sex, ethnicity, education level, occupational position, alcohol consumption, smoking status, physical activity, BMI, systolic blood pressure, diastolic blood pressure, glucose, total cholesterol, and triglycerides, high-density lipoprotein cholesterol, anti-hypertensive drugs, diabetes medication, and lipid lowering drugs. X axis for high-sensitivity troponin I was log2 transformed. Y axis presents hazard ratio on the logarithmic scale.


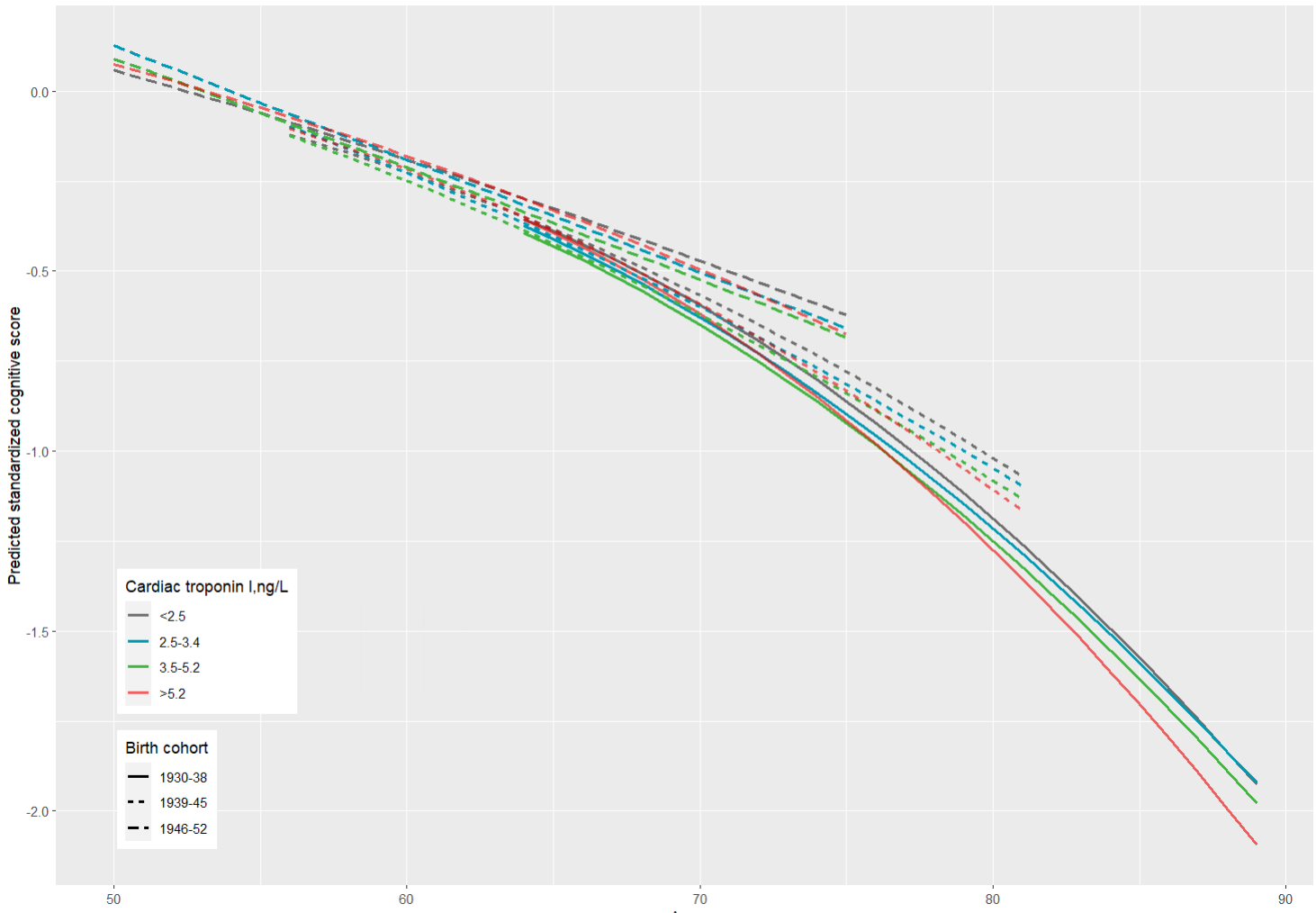


Figure S2 Cognitive trajectories from age 50 to 89 years by levels of high-sensitivity cardiac troponin I at baseline stratified by birth cohort. Predicted cognitive scores estimated from a mixed model (model terms: sex, ethnicity, education level, occupational position, birth cohort, cardiac troponin group, age, age2, and interactions of sex, ethnicity, education level, occupational position, birth cohort, cardiac troponin group with age and age2, alcohol consumption, smoking status, physical activity, BMI, SBP, DBP, glucose, ~~and~~ total cholesterol, triglycerides, high-density lipoprotein cholesterol, anti-hypertensive drugs, diabetes medication, and lipid lowering drugs). P for difference in trajectory between different cardiac troponin levels: 0.0013


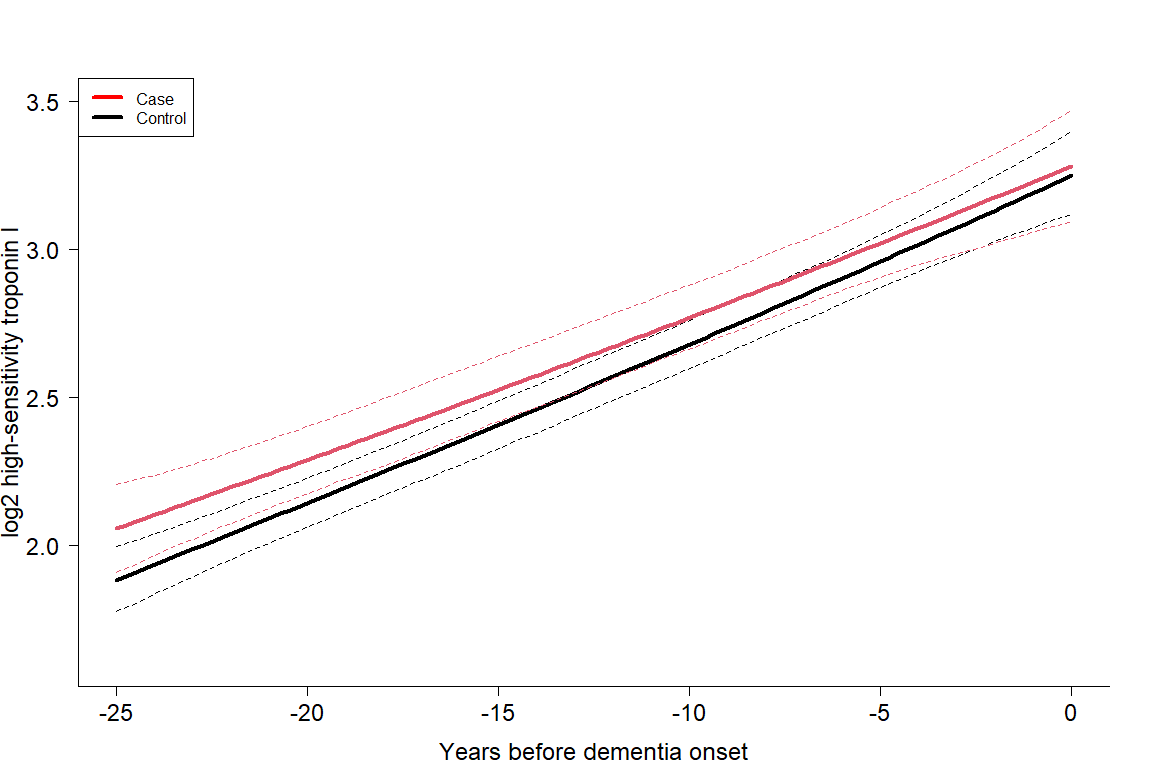


Figure S3 Averaged predicted trajectories of high-sensitivity cardiac troponin I (ng/L, log2 transformed) for 691 incident cases of dementia and 2764 matched controls over 25 years preceding diagnosis of dementia estimated from a latent process mixed model which included time, time2, index age (age at time 0), sex, education level, case indicator (coded as 1 for cases and 0 for controls), and interactions of all the above covariates (index age, sex, education level, and case indicator) with time and time2. Nested case-control design matching with age, sex and education level and cardiovascular disease status. Dashed lines represent 95% CIs. P for difference in trajectory between cases and controls: 0.07


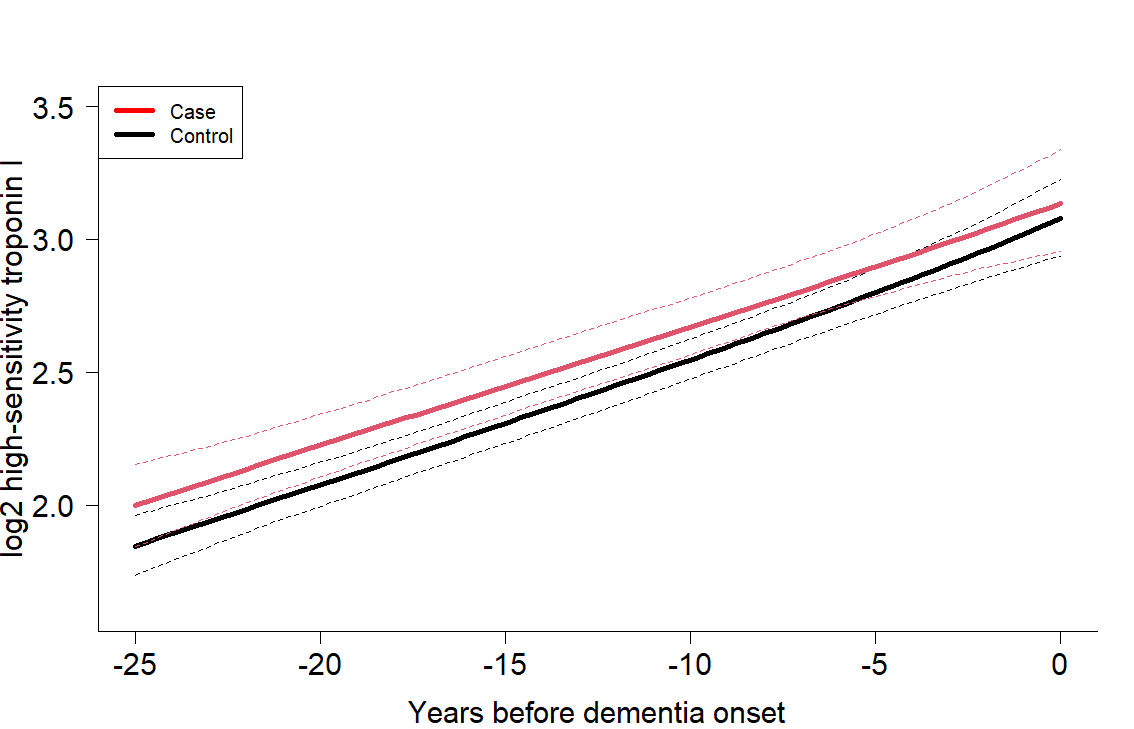


Figure S4 Averaged predicted trajectories of high-sensitivity cardiac troponin I (ng/L, log2 transformed) for 695 incident cases of dementia and 2780 matched controls over 25 years preceding diagnosis of dementia estimated from a latent process mixed model which included time, time2, index age (age at time 0), sex, education level, case indicator (coded as 1 for cases and 0 for controls), and interactions of all the above covariates (index age, sex, education level, and case indicator) with time and time2, time-dependent SBP, fast glucose, BMI, total cholesterol, triglycerides, and HDL. Nested case-control design matching with age, sex and education level. Dashed lines represent 95% CIs. P for difference in trajectory between cases and controls: 0.34
